# Postmortem brain 7T MRI with minimally invasive pathological correlation in deceased COVID-19 subjects

**DOI:** 10.1186/s13244-021-01144-w

**Published:** 2022-01-15

**Authors:** Maria da Graça Morais Martin, Vitor Ribeiro Paes, Ellison Fernando Cardoso, Carlos Eduardo Borges Passos Neto, Cristina Takami Kanamura, Claudia da Costa Leite, Maria Concepcion Garcia Otaduy, Renata Aparecida de Almeida Monteiro, Thais Mauad, Luiz Fernando Ferraz da Silva, Luiz Henrique Martins Castro, Paulo Hilario Nascimento Saldiva, Marisa Dolhnikoff, Amaro Nunes Duarte-Neto

**Affiliations:** 1 Instituto de Radiologia, Hospital das Clinicas HCFMUSP, Faculdade de Medicina, Universidade de São Paulo, LIM44, Travessa da Rua Dr. Ovídio Pires de Campos, 75, São Paulo, SP 05403-010 Brazil; 2 Departamento de Patologia, Faculdade de Medicina FMUSP, Universidade de São Paulo, São Paulo, Brazil; 3 Neurology Department, Faculdade de Medicina FMUSP, Universidade de São Paulo, São Paulo, Brazil; 4 Instituto Adolfo Lutz, São Paulo, Brazil; 5 Departamento de Radiologia e Oncologia, Faculdade de Medicina, Universidade de São Paulo, LIM 44 HCFMUSP, São Paulo, Brazil; 6 Departamento de Patologia, Faculdade de Medicina FMUSP, Universidade de São Paulo, Servico de Verificaçao de Óbitos de São Paulo (SVO), São Paulo, Brazil

**Keywords:** COVID-19, Neuroimaging, MRI, Autopsy, Neuropathology

## Abstract

**Background:**

Brain abnormalities are a concern in COVID-19, so we used minimally invasive autopsy (MIA) to investigate it, consisting of brain 7T MR and CT images and tissue sampling via transethmoidal route with at least three fragments: the first one for reverse transcription polymerase chain reaction (RT-PCR) analysis and the remaining fixed and stained with hematoxylin and eosin. Two mouse monoclonal anti-coronavirus (SARS-CoV-2) antibodies were employed in immunohistochemical (IHC) reactions.

**Results:**

Seven deceased COVID-19 patients underwent MIA with brain MR and CT images, six of them with tissue sampling. Imaging findings included infarcts, punctate brain hemorrhagic foci, subarachnoid hemorrhage and signal abnormalities in the splenium, basal ganglia, white matter, hippocampi and posterior cortico-subcortical. Punctate brain hemorrhage was the most common finding (three out of seven cases). Brain histological analysis revealed reactive gliosis, congestion, cortical neuron eosinophilic degeneration and axonal disruption in all six cases. Other findings included edema (5 cases), discrete perivascular hemorrhages (5), cerebral small vessel disease (3), perivascular hemosiderin deposits (3), Alzheimer type II glia (3), abundant *corpora amylacea* (3), ischemic foci (1), periventricular encephalitis foci (1), periventricular vascular ectasia (1) and fibrin thrombi (1). SARS-CoV-2 RNA was detected with RT-PCR in 5 out of 5 and IHC in 6 out 6 patients (100%).

**Conclusions:**

Despite limited sampling, MIA was an effective tool to evaluate underlying pathological brain changes in deceased COVID-19 patients. Imaging findings were varied, and pathological features corroborated signs of hypoxia, alterations related to systemic critically ill and SARS-CoV-2 brain invasion.

## Key points

- Minimally invasive autopsy was an effective tool to evaluate underlying pathological brain changes in deceased COVID-19 patients.
- Imaging findings were varied, and pathological features corroborate signs of hypoxic injury, alterations related to systemic critically ill and SARS-CoV-2 brain invasion
- SARS-CoV-2 RNA was detected with RT-PCR and with immunohistochemistry even in the samplings of brains with normal postmortem MRI.

## Background

COVID-19 has dramatically evolved from a mysterious pneumonia to global pandemic of a multisystem disease with more than five million deaths worldwide so far.

Neuropsychiatric symptoms are an important concern in COVID-19 patients. Neurological symptoms in COVID-19 include headache, dizziness, seizures, anosmia, ageusia, focal deficits (quadriparesis, hemiparesis, aphasia), confusion and post-extubation delayed awakening. Post-COVID neuropsychiatric complains are also frequent. Disease mechanisms, however, remain incompletely understood. The proposed mechanisms include direct nervous tissue viral aggression, host response, hypoxia, stroke, critically ill patients’ related injuries, treatment side effects or a combination of these factors.

Since Renaissance, autopsy has been used as a tool to investigate disease pathophysiology. In a pandemic scenario, however, exposure of autopsy teams to a highly contagious agent must be avoided.

In this context, our service has performed ultrasound-guided minimally invasive autopsies (MIA-US) to obtain tissue samples from several organs, while reducing risks of contamination [1]. Since ultrasound is limited to evaluate the adult brain, we associated 7T magnetic resonance imaging (MRI), computed tomography (CT) and transethmoidal brain sampling to further investigate SARS-CoV-2 brain-related injuries.

The aim of the present study is to describe imaging and histopathological findings in deceased COVID-19 patients whose brains were assessed with this approach.

## Material and methods

In the current COVID-19 pandemic, Hospital das Clínicas da Faculdade de Medicina da USP (HCFMUSP), a tertiary teaching public hospital in the city of São Paulo, Brazil, was assigned as the reference service to treat severely ill COVID-19 patients. Severe illness was defined as acute respiratory distress syndrome with computed tomography characteristic findings of COVID-19 involvement, hypoxemia requiring supplementary oxygen (e.g., nasal catheter or mechanical ventilation) and patients with comorbidities. Cases were confirmed with SARS-CoV-2 RNA detection by reverse transcriptase–polymerase chain reaction (RT-PCR) in respiratory samples, following the Charité protocol, validated in our institution [1, 2].

Autopsies were performed in the Pathology Department of Faculdade de Medicina da Universidade de São Paulo and in the “Image Platform in the Autopsy Room” (PISA) facilities (https://pisa.hc.fm.usp.br/) in the same institution, after written consent from the first‐degree relative. Data collection was performed in April and May of 2020. Epidemiological, clinical and laboratory data were collected from relatives and medical records. This work and protocol were approved by HCFMUSP Ethical Committee (protocol #3951.904).

Postmortem brain MRI was acquired in a 7T Siemens Magnetom scanner (Siemens, Erlangen, USA) with a 32-channel coil (Nova Medical, Wilmington, USA). Corpses were wrapped in appropriate plastic bags before entering the scanner. 3DMP2RAGE (TE 1.9 ms, TR 6000 ms, 0.75 mm isotropic voxels), coronal T2 (TE 60 ms, TR 4170 ms, 0.23 mm in plane resolution, 2 mm thickness), axial T2 (TE 61 ms, TR 7000 ms, 0.45 mm in plane resolution, 2 mm thickness), FLAIR (TE 82 ms, TR 7100 ms, 0.55 mm in plane resolution, 3.5 mm thickness), DWI and SWI (TE 14 ms, TR 23 ms, 0.2 mm in plane resolution, 1.3 mm thickness) images were acquired. A total body CT image was also acquired before MRI, and US-guided tissue sampling was performed after MRI. MR and CT images were analyzed by two independent neuroradiologists (M.G.M.M. and E.F.C.) and reviewed for consensus.

**Fig. 1:**
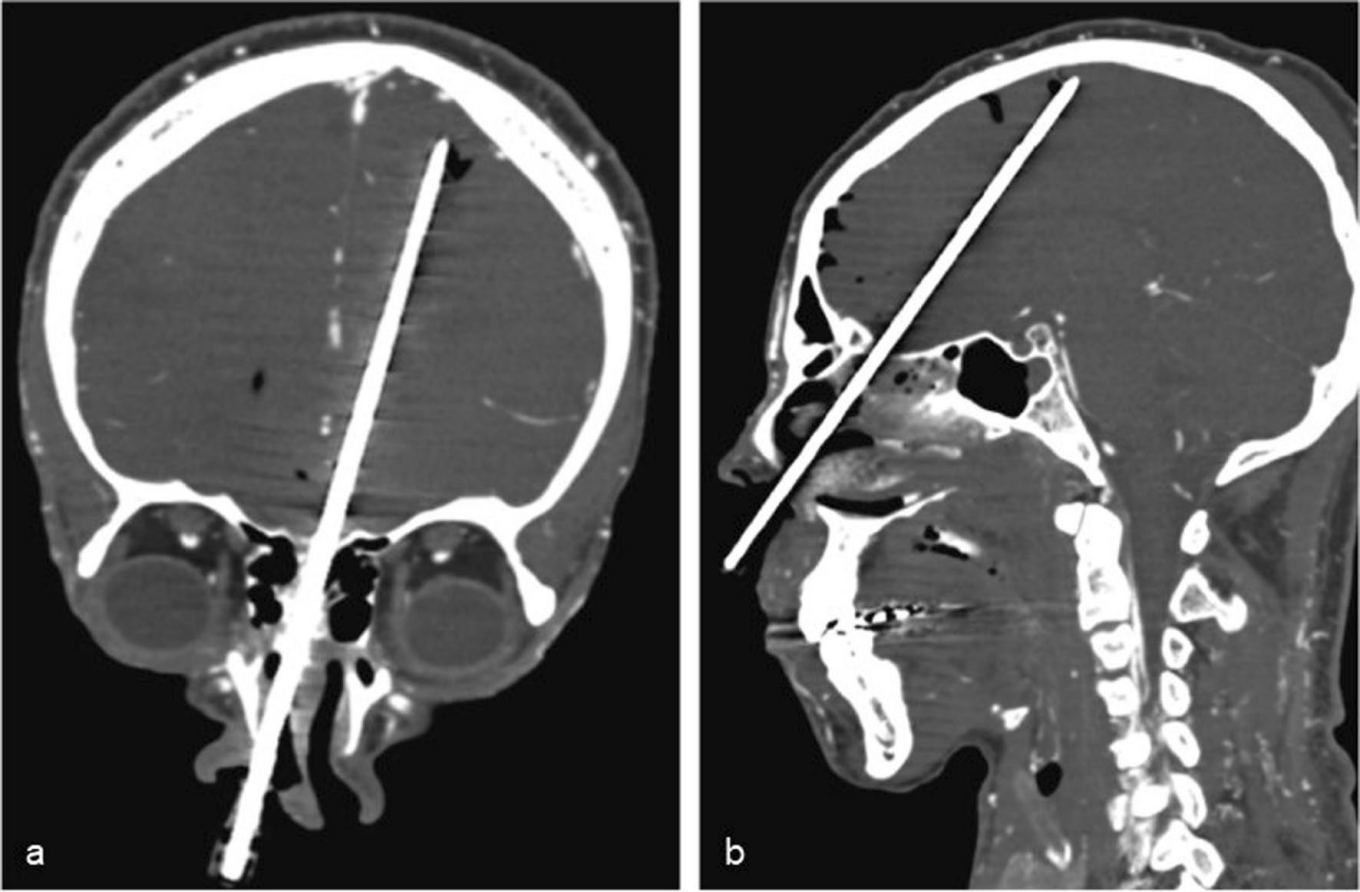
Coronal (**a**) and sagittal (**b**) CT reconstructions illustrating the Tru-Cut needle during a transethmoidal brain tissue sampling

Brain sampling was performed via transethmoidal route (Fig. 1), using Tru-Cut® semiautomatic coaxial needles of 14G, 20 cm long. At least three fragments were obtained in each case. The first fragment was stored at -80 °C and used for RT-PCR analysis [1, 2]. The other fragments were fixed in 10% buffered formalin, embedded in paraffin and stained with hematoxylin and eosin (H&E). Although the same region was attempted, angle of access might have changed a little between the subjects. Histological analysis was performed by two independent pathologists (A.N.D.N. and V.R.P.), with posterior consensus agreement. Immunohistochemical (IHC) reactions were performed using an anti-SARS-CoV-2 nucleocapsid protein (mouse monoclonal antibody [6H3] GeneTex cat. GTX-632269, GeneTex Inc., Irvine, CA, USA, 1:500 dilution) and anti-SARS-CoV-2 Spike S2 protein (MP Biomedicals, code 08720402, Irvine, Califórnia, USA, 1:500 dilution). The antigen retrieval was performed with 10 mM citrate buffer pH 6.0. IHC reactions were amplified by alkaline phosphatase conjugated polymers (Polink AP Broad, GBI Labs, Bothell, WA, USA) and revealed with fast red chromogen (GBI Permanent Red, GBI Labs, Bothell, WA, USA). The primary antibodies were first tested in lung samples from patients with COVID-19 pneumonia, and negative controls included tissue samples of brains from patients with cardiovascular diseases and other viral infections, obtained from our autopsy archive. The IHC reactions resulted negative in all the negative controls.

## Results

Minimally invasive autopsies with brain MRI were performed in 7 deceased COVID-19 patients, with brain tissue sampling in 6 of them. (Patient 3’s family did not consent to cerebral tissue sampling.) Table 1 displays the main clinical, radiological and pathological encountered features. One case (case 4) was previously described, focusing on cardiac complications attributed to COVID-19 [3] and on the spectrum of severe COVID-19 in children [4]. COVID-19 infection was confirmed in all cases by positive SARS-CoV-2 RNA detection in naso/oropharyngeal swabs, collected antemortem in 5 cases, and postmortem in 2. All cases disclosed typical COVID-19 lung CT findings, with greater than 50% percent lung involvement. Mean age was 44 years (ranging from 11 to 74 years), 4 were women, one of whom was in the third trimester of pregnancy. Mean body mass index was 28.7 (15.2–48.4). One patient had acute neurological symptoms (case 2), presenting with new onset epileptic seizures (previous history of depression). Among the other patients, three presented headaches as an initial symptom. Mean D-dimer was 16,308 ng/mL FEU (1,023—1200,881 ng/mL FEU, reference range < 500 ng/mL FEU), and C reactive protein was 146 mg/L (49–240 mg/L, reference range < 5,0 mg/L). All patients were very critically ill, with acute respiratory distress syndrome and mechanical ventilation, associated with acute kidney failure and refractory shock. One patient received thrombolytic treatment (case 3), and two received anticoagulants (cases 3 and 7). Death was attributed to severe COVID-19 in all cases.

**Table 1:** Clinical, radiological and pathological central nervous system features in seven fatal COVID-19 cases

Patient demographics	Clinical features	Time from symptom onset to death (days)	Length MV (days)	Head CT	Head MRI	Brain histology
Patient 1 M, 45y	Heart failure, anasarca, dyspnea, ARDS, ARF	12	5	Bilateral old infarcts (right frontal and left parietal)	Bilateral cortical infarcts (right frontal and left parietal) with cortical necrosis and petechial hemorrhage (probably chronic) and old left cerebellar infarct (Fig. 2)	Reactive microglia, "red neurons," edema, congestion, fibrin thrombi, perivascular hemorrhages and hemosiderin deposits, small vessel disease RT-PCR + IH +
Patient 2 F, 39y	Anasarca, abdominal pain, nausea, dyspnea, ARF, seizures. Cardiorespiratory arrest, shock, thrombolytic therapy in the day of death due to suspected pulmonary embolism. Previous depression	31	2	Subarachnoid hemorrhage, bilateral basal ganglia and parietal hypoattenuation	Subarachnoid hemorrhage, corticospinal tract, basal ganglia and cortical subcortical biparietal and right frontal signal abnormalities (Fig. 3)	Reactive microglia, "red neurons," Alzheimer type II glia, edema, congestion, perivascular hemorrhages and hemosiderin deposits RT-PCR + IH +
Patient 3 M, 74y	SAH, DM, ischemic cardiopathy, Flu-like symptoms (myalgia, cough, adynamia, poor appetite), ARDS, stupor, A-V block, cardiorespiratory arrest, refractory shock	17	10	Diffuse cerebral edema	Diffuse cerebral edema and patchy white matter focal abnormalities (Fig. 4a, b)	Not evaluated *
Patient 4 F, 11y	Flu-like symptoms, odynophagia, fever, chest pain, headache, diarrhea, ARDS, AKI, refractory shock	8	1	Normal	Focal signal abnormality in the splenium of corpus callosum with microbleeds and subtle sulcal effacement (Fig. 4c, d)	Reactive microglia, "red neurons," edema, congestion, discrete perivascular hemorrhages IH + (RT-PCR not performed)**
Patient 5 F, 74y	Breast cancer (2019 treatment), neutropenia, SAH, hypothyroidism, cough, fever, ARDS, ARF, pneumothorax (drained) and refractory shock	23	3	Atheromatosis, globus pallidus calcification, focal areas of white matter hypoattenuation	Focal white matter signal abnormalities and focal frontal white matter hemorrhagic foci (Fig 5a, b)	Reactive microglia, "red neurons," edema, congestion, perivascular hemorrhages, small vessel disease and focal ischemia RT-PCR + IH +
Patient 6 F, 35y	Pregnancy (third trimester), asthma, DM, obesity, myalgia, chills, headache, fever, anosmia and ageusia, dyspnea, ARDS, severe bronchospasm, emergency cesarean section, tachyarrhythmias, CPR > 1 h	16	7	Normal	Normal	Reactive microglia, "red neurons," congestion, periventricular vascular ectasia, small vessel disease, Alzheimer type II glia RT-PCR + IH +
Patient 7 M, 32y	Flu-like symptoms, fever, headache, diarrhea, myalgia, decreased appetite and cough. ARDS, pneumonia, ECMO, refractory shock	32	7	Normal	Few punctate hemorrhagic periventricular foci (Fig 5c, d)	Reactive microglia, "red neurons," edema, congestion, perivascular hemorrhages, Alzheimer type II glia, peri-ependymal focal hemorrhagic encephalitis with mixed inflammatory reaction RT-PCR + IH +

A-V = atrial ventricular ARF = acute renal failure; ARDS = acute respiratory distress syndrome; CPR = cardiopulmonary resuscitation**;** DM = diabetes mellitus; ECMO = extracorporeal membrane oxygenation; HF = heart failure; IH = immunohistochemistry; RT-PCR = real-time polymerase chain reaction; SAH = systemic arterial hypertension

Immunohistochemistry was positive in the cytoplasm of parenchymal endothelial cells and scattered microglial cells

*Family did not consent to cerebral tissue sampling for this patient

**Case 4 fresh frozen brain sample not collected for RT-PCR

**Fig. 2:**
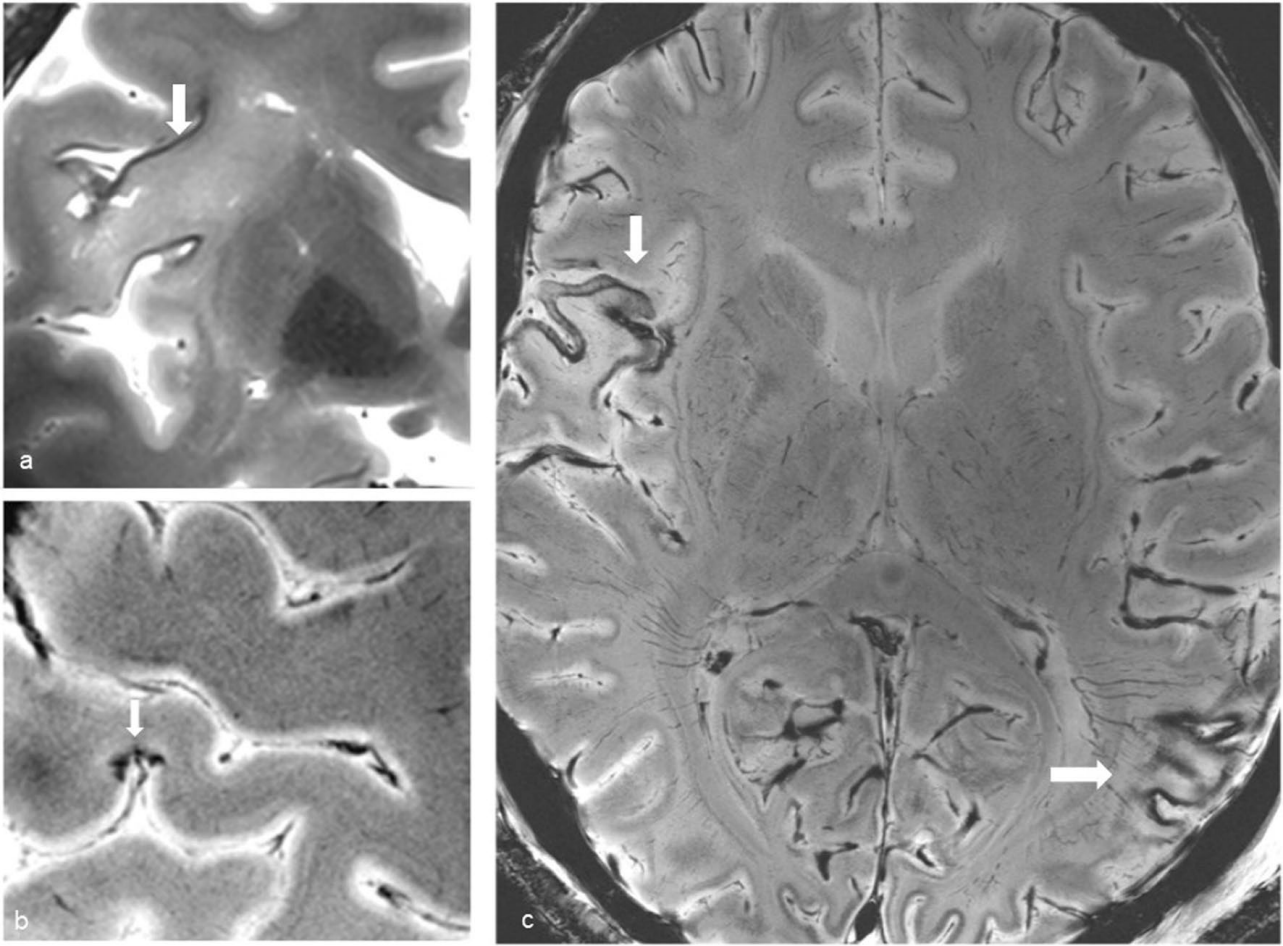
Coronal T2 (**a**) and axial SWI (**b**, **c**) of patient 1 showing two large (right frontal and left parietal) cortical–subcortical infarcts with petechial cortical hemorrhage, and a smaller right frontal cortical infarct (**b**). Previous brain imaging unavailable, so timing of the infarct could not be established; imaging features do not indicate an acute lesion; previous heart failure could favor chronic lesions

**Fig. 3:**
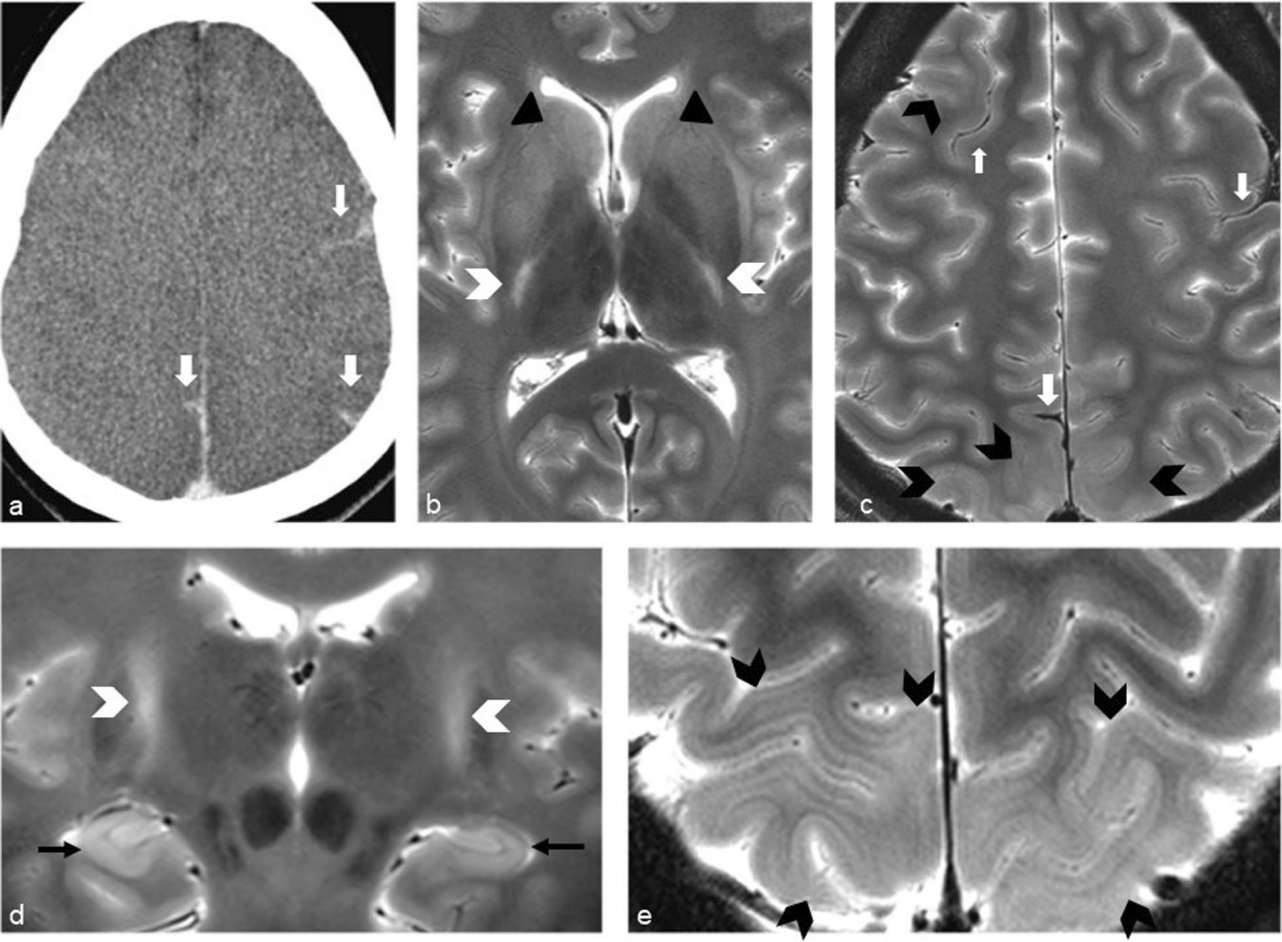
Patient 2. Bilateral subarachnoid hemorrhages (white arrows) in frontal and parietal sulci on CT (**a**) and axial T2 (**c**), signal abnormalities in the corticospinal tracts (white arrowheads **b**, **d**), bilateral basal ganglia (**b**—black arrowheads), hippocampi (**d**—black arrows), bilateral cortical/subcortical parietal lobes, and more subtle in the right frontal lobe (**c**, **e**—black arrowheads)

**Fig. 4:**
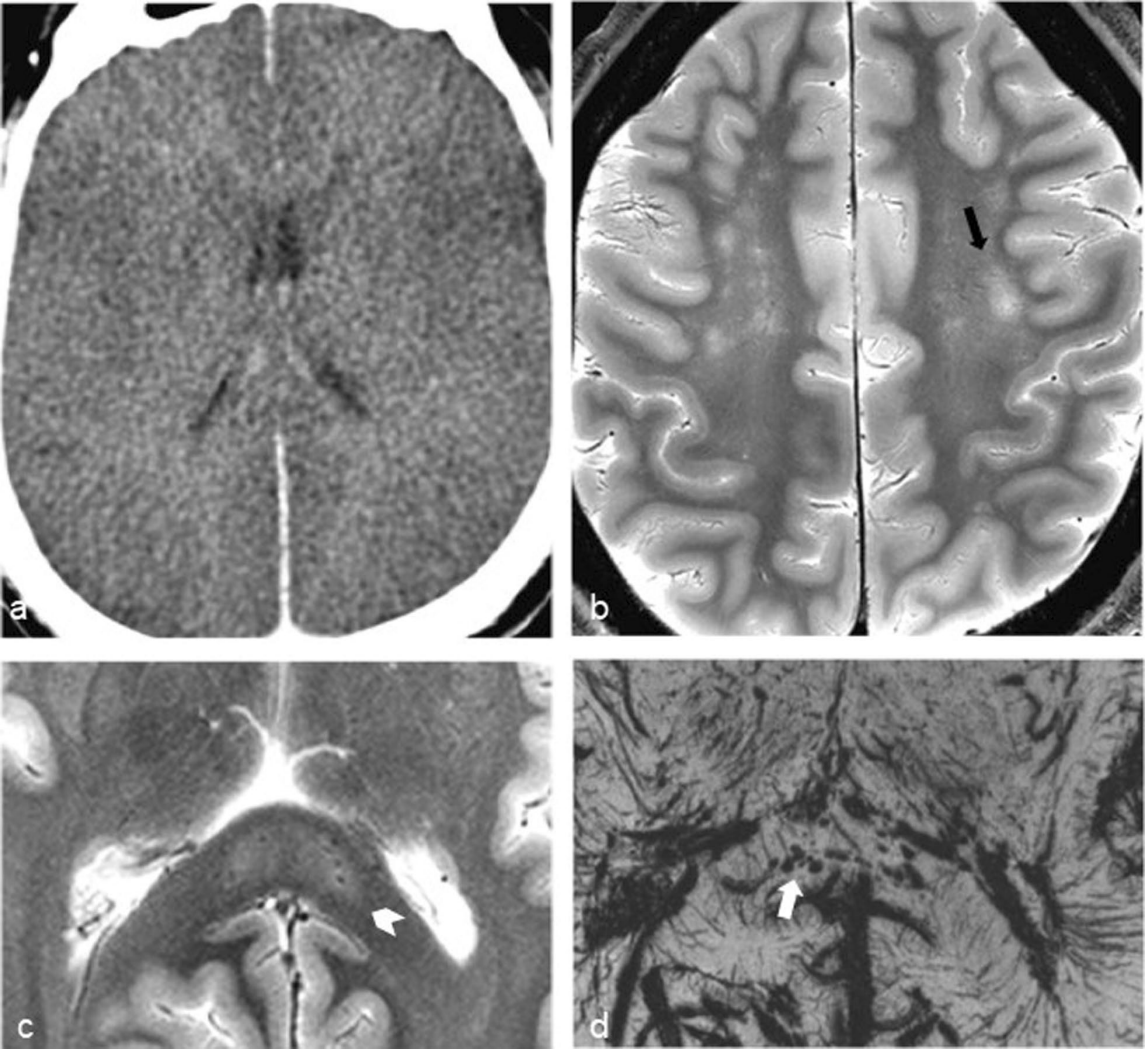
Diffuse edema (CT **a**) and some patchy white matter focal abnormalities (axial T2 **b**) (black arrow) in patient 3. Patient 4 had splenial signal abnormality (white arrowhead) with high T2 signal (**c**) with punctate hemorrhagic foci on SWI (**d**) (white arrow)

**Fig. 5:**
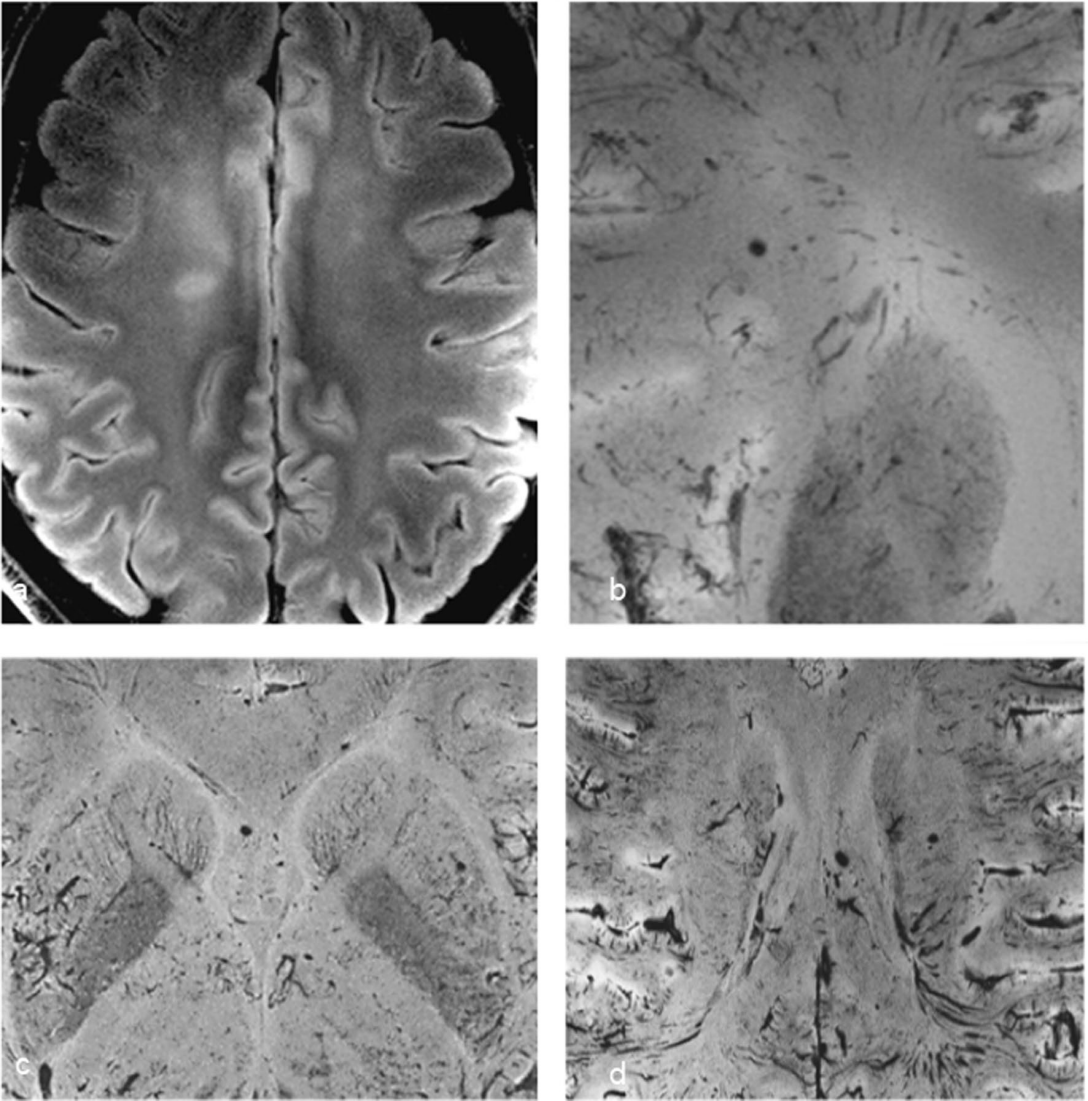
Nonspecific multifocal white matter abnormalities with increased signal on FLAIR (**a**) and punctate hemorrhagic foci in the right white matter on SWI (**b**) of patient 5. Patient 7 showed some periventricular punctate hemorrhagic foci on SWI (**c**, **d**)

Brain imaging (CT and MRI) studies were normal in one subject (patient 6) and abnormal in the other six. One patient had cortical–subcortical probably chronic infarcts with petechial hemorrhages (two large infarcts—right frontal and left parietal, and two smaller infarct—left cerebellar and right frontal) (Fig. 2). One patient had bilateral frontal and parietal subarachnoid hemorrhage, and signal abnormalities in both corticospinal tracts, bilateral basal ganglia, hippocampi and cortical/subcortical parietal bilaterally, and in the right frontal lobe (Fig. 3). Increased T2 and FLAIR signal associated with punctate hemorrhagic foci was identified in the splenium of corpus callosum in patient 4 (Fig. 4c, d). Patient 5 had some nonspecific multifocal white matter abnormalities with increased T2 and FLAIR signal, and some punctate hemorrhagic foci in the right frontal white matter (Fig. 5a, b). Periventricular punctate hemorrhagic foci were noted in patient 7 (Fig. 5c, d). One subject had diffuse edema and patchy white matter focal abnormalities. This patient did not undergo cerebral sampling (family did not consent) (Fig. 4a, b).

**Fig. 6:**
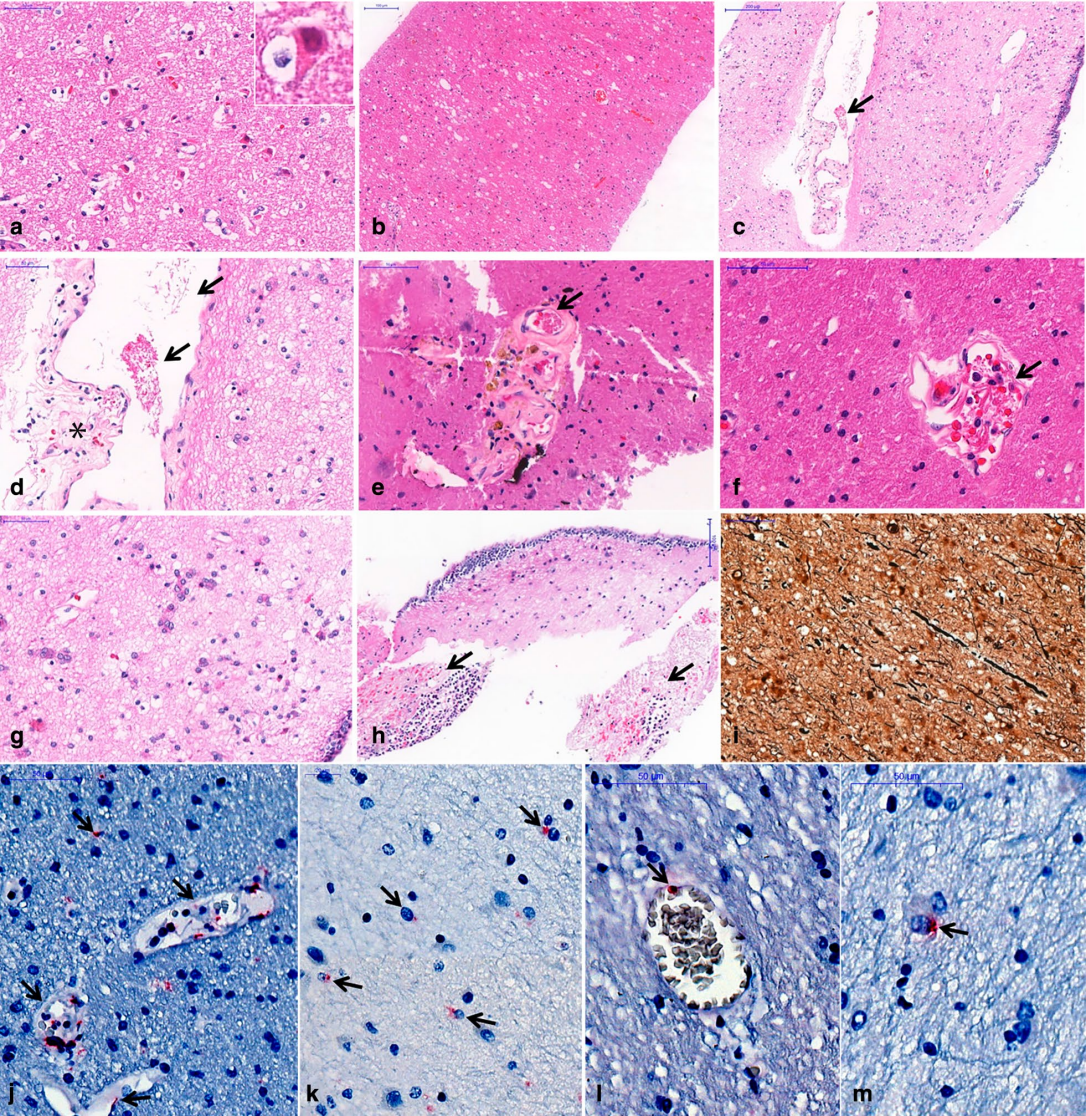
Histological findings in the brain in six fatal COVID-19 cases. **a** Eosinophilic degeneration of cortical neurons (“red neurons”) secondary to hypoxia; **b** white matter edema and congestion; **c**, **d** periventricular vessel with small fibrin clot (arrows), perivascular edema and red cells perivascular leakage (asterisk); **e** fibrin clot within (arrow) a vessel with lipohyalinosis and perivascular hemosiderin deposits, compatible with previous small vessel disease and cerebral microhemorrhages; **f** fibrin within a vessel with perivascular edema and discrete perivascular bleeding (arrow); **g** Alzheimer type II glia in periventricular area; **h** foci of periventricular neutrophilic infiltrate and recent hemorrhages (arrows); **i** Bielschowsky's silver stain revealing axonal disruption and thickening. **j**–**m** Immunohistochemistry reaction detected SARS-CoV-2 nucleocapsid protein in the cytoplasm of endothelial cells (**j**,** l**) and microglial cells (**k**,** m**), (arrows), (alkaline phosphatase).** a–h**: H&E stain

Histological brain analysis showed reactive gliosis, congestion and cortical neuron eosinophilic degeneration, and axonal disruption in all six cases (Fig. 6). Other findings included edema (5 cases), discrete perivascular hemorrhages (5), cerebral small vessel disease (3), perivascular hemosiderin deposits (3), Alzheimer type II glia (3), abundant corpora amylacea (3), foci of ischemic area (1), foci of periventricular encephalitis (1), fibrin thrombi (1) and periventricular vascular ectasia (1) (Fig. 6). IHC reactions were positive with the two primary antibodies, labeling endothelial cells and microglial cell in 6 out of 6 patients (Fig. 6). SARS-CoV-2 RNA was detected by RT-PCR in the brain specimens in 5 out of 5 cases (100%). Two specimens were not evaluated (patient 3 did not have tissue sampled, as previously explained, and patient 4 did not have frozen tissue). Other autopsy findings included typical COVID-19 pneumonia (diffuse alveolar damage, pneumocyte cytopathic changes, fibrin thrombi and increased megakaryocytes in septal vessels), acute tubular necrosis and visceral congestion.

## Discussion

We showed brain postmortem high-resolution images of COVID-19-deceased patients, with histological correlations.

Cerebrovascular abnormalities were the most common findings, detected in five patients: one stroke (although timing was not established), one subarachnoid hemorrhage (although she had also undergone thrombolytic therapy for pulmonary embolism) and three patients with microhemorrhage. They are the most commonly described CNS imaging finding in COVID-19 patients [5], and potential mechanisms include endothelial damage linked to the angiotensin-converting enzyme 2 (ACE2) receptor, hypercoagulable state and cardiomyopathy [7].

A recent meta-analysis of 108,571 patients with COVID-19 [9] showed that acute cerebrovascular disease occurred in 1.4%, with acute ischemic stroke accounting for 88% of those and intracerebral hemorrhage for 12%. They were more frequent in older subjects, in those who were severely infected and had preexisting vascular risk factors. Compared to individuals who experienced a stroke without the infection, patients with COVID-19 and stroke were younger, had higher National Institutes of Health Stroke Scale/Score (NIHSS), higher frequency of large vessel occlusion and higher in-hospital mortality rate. The pattern of large vessel occlusion and multi-territory infarcts suggests that cerebral thrombosis and/or thromboembolism could be possible causative pathways for the disease. Hemorrhage could be associated with arterial wall damage and rupture related to SARS-CoV-2 affinity for ACE2 receptors, which are expressed in brain endothelial and arterial smooth muscle cells. Alternatively, hemorrhagic infarcts could be related to the inflammatory response [8].

Intra-axial susceptibility abnormalities suggestive of microhemorrhage were the most common finding (74%) in a consecutive cohort of COVID-19 patients [10] and have also been reported in postmortem brain analysis in COVID-19 patients [11]. Nonetheless, these abnormalities have also been described in critically ill patients, patients using extracorporeal membrane oxygenation (ECMO) and in high-altitude hypoxia, and are not, therefore, specific to COVID-19. In our samples, microvascular brain damage was detected in three cases on MRI (and in 5 cases in pathology, 3 of them being the ones with MRI findings). Pathological analysis was superior to imaging in detecting perivascular hemorrhages, possibly due to higher spatial resolution (pathologically detected microhemorrhages were very small and adjacent to the vessel, and together, they were smaller than the in-plane resolution of the SWI acquisition (0.20 mm), making it impossible to differentiate one another: For example, in Fig. 6e there is hemosiderin adjacent to the vessel, and together, they measure 0.15 mm; in Fig. 6f, the vessel and adjacent hemorrhage measure 0.10 mm). Besides this issue, the SWI artifact related to blood products is usually bigger than the blood products themselves, being another explanation why these small perivascular hemorrhages could not be differentiated from the vessels in the MRI. This is particularly a problem in the in situ postmortem brain 7T MRI, because the intravessel susceptibility signal (from postmortem intravessel stasis/thrombi) is very strong in this scenario, as shown in Fig. 4d for example. Although a previous paper showed underestimation of CMBs by postmortem MRI brain compared to histology, in up to 24%[12], another study using 7T showed better correlation with MRI, especially for those in the cortico-subcortical regions (using ex situ images, which have much smaller intravessel SWI, because much of the blood has been washed away during fixation). Another issue is that, besides the resolution, MRI might also show false positives. The same study using 7T MRI and postmortem histology showed that in the deep white matter 42% of microhemorrhages were not detected, while 31% of T2* hyposignals were not due to microhemorrhages, but due to vessels filled with postmortem thrombi [13], adding even more nuances to this diagnosis.

One patient (patient 2) had other imaging findings besides hemorrhage. Posterior cortical–subcortical abnormalities could represent posterior reversible encephalopathy (PRES), which have been described in COVID-19-deceased patients [14], and possibly related to endothelial cell damage due to viral ACE receptor binding. Clinical features were also compatible with PRES, that is commonly associated with seizures. An alternative explanation for these imaging findings would be hypoxic changes. Imaging findings in the basal ganglia could represent PRES, encephalitis, metabolic-related changes or hypoxic injury. One of the sampled fragments included part of the basal ganglia. Histological analysis of that fragment did not disclose any evidence of encephalitis, such as perivascular infiltrates, glial nodules or cytopathic changes. Most of the histological changes in this case appear to represent systemic illness responses and hypoxic injury. Hippocampal changes could also be related to hypoxia. Clinical history corroborates hypoxic injury, since, in addition to prolonged mechanical ventilation related to hypoxia, the patient also suffered four episodes of cardiorespiratory arrest during hospitalization. Corticospinal tract signal abnormalities have not been previously described in COVID-19 patients, although paraparesis has been reported. Wallerian degeneration resulting from cortical hypoxia is a possible explanation. These findings were supported by axonal thickening and disruption on Bielschowsky stain. The axonal lesion (so-called Wallerian-like degeneration) is well described and associated with ischemic lesion in experimental and clinical studies [15, 16]. Although it can be difficult to distinguish from traumatic brain lesion, the other histopathological changes and the clinical context can help in differential evaluation [16, 17]. A direct point-to-point correlation between imaging and histology findings was not possible, limiting interpretation of imaging and pathology findings.

A focal lesion in the splenium of the corpus callosum was identified in patient 4, which may occur in diverse clinical scenarios, such as epilepsy, encephalitis and drug-related, and has been described in COVID-19 patients [18], especially in children with MIS-C (multisystem inflammatory syndrome in children) [19]. These lesions are usually transitory and are characterized by increased T2 and FLAIR signal, with diffusion restriction. Some authors propose that cytokine‐mediated edema may underlie this finding [20] which would be in agreement with this prevalence in children with MIS-C. Hemorrhagic foci, such as seen in this case, are not frequently associated with these transitory lesions, but have been previously described in COVD-19 patients [21].

Finally, white matter hyperintense lesions were detected in two patients (Patients 3 and 5). The lesions usually represent a nonspecific finding, which may be related to chronic white matter small vessel disease. Acute white matter abnormalities have been reported in COVID-19 patients [22], especially centrum semi-ovale lesions showing restricted diffusion, sometimes associated with globus pallidus lesions [23], representing either ADEM-like lesions or, most likely, ischemic/vasculitic lesions [22, 23]. In one case, tissue sampling was not performed, and in the other one, histological analysis showed chronic small vessel changes. Small vessel disease is a common finding in patients with hypertension and diabetes, underscoring the relevance of preexisting conditions as risk factors for an unfavorable outcome.

Previously reported imaging findings in COVID-19 patients, such as meningeal enhancement (that cannot be adequately assessed by postmortem studies), gyral swelling and diffuse white matter signal abnormalities were not detected in our series [10].

Regarding the clinical scenario, all patients were critically ill, in the intensive care unit, with respiratory failure requiring mechanical ventilation, associated with renal failure evolving to refractory shock. Headache, a commonly described complaint, was reported by three subjects as initial symptoms. One patient, who showed the most prominent abnormal imaging finding, presented with new onset seizures leading to hospital admission.

Of all the patients in our series, only one underwent in vivo brain imaging (normal head CT). This is a limitation of this study. Corresponding in vivo imaging would be ideal to better differentiate postmortem aspects from in vivo findings. Typical postmortem brain findings and challenges include: different T1, T2 and ADC due to a combination of factors like lower temperature and decomposition (that can be affected by postmortem interval, ambient, etc.); loss of grey–white matter differentiation mainly on CT, but with some blurring on MRI, due to fluid shift and cerebral autolysis; drastically different susceptibility-weighted image due to a darker vessel signal mainly related to blood stasis (which is even more exacerbated in 7T imaging); gas appearing linked to putrefaction; no intravenous contrast administration (although postmortem angiography might be done—in which case brain enhancement might be normally expected) [24]; hyperdensity (or clot signal on MRI) of the dural venous sinuses, sometimes in a dependent position; increase in brain volume and loss of definition of sulci, which may be due to a combination of vasogenic and cytotoxic edema and may be more pronounced in death with prolonged duration of the agonal state, leading to a longer hypoxic state, than in acute death [25, 26]; and in more advanced deterioration “softening” of brain tissue and settling of the tissue against the dependent part of the skull [27–30]. Obtaining in vivo neuroimaging, especially MRI, in critically ill COVID-19 patients, with high infectious potential is limited, leading to underestimation of neurological involvement in these patients.

The pathological analysis of our samples showed reactive gliosis, congestion and cortical neuron eosinophilic degeneration, axonal disruption and the presence of SARS-CoV-2 antigens in the endothelial cells and microglia and RNA detection in all analyzed cases. Edema was identified in 5 cases (perivascular scarce neuropil, forming “vacuoles”), exudation of fibrin and hemosiderin (meaning that an extravascular leak of red cells has occurred, days before the death) (Fig. 6c–f). This is a most probably diffuse multifactorial pathological process, involving the endothelial cells, leading to extravascular leakage: SARS-CoV-2 infection of endothelial cells, as we show positive detection of the N-antigen in the cytoplasms of those cells (Fig. 6j, l); hypoxia and shock. Alzheimer type II astrocyte was identified in 3 patients. It represents a common change in (and an important marker for) patients with metabolic impairments, notably renal and hepatic failure [31, 32]. Although their origin and relation to clinical manifestations is unclear, some studies (specially experimental ones) propose that these cells arise in the same pathophysiological background as the neurological manifestations (changes in glutamine and ammonia levels) [33]. Only one case showed mixed periventricular inflammatory infiltrates (Patient 7). Neuropathological studies on COVID-19 are scarce so far. One case report disclosed findings suggestive of both vascular and demyelinating processes [34]. Another study included 18 brains from deceased COVID-19 patients [35] and showed mainly hypoxic injury in the cerebrum and cerebellum, with neuronal loss in the cerebral cortex, hippocampus, and cerebellar Purkinje cell layer. These findings are concordant with ours. In two cases, rare foci of perivascular lymphocytes were detected, and leptomeningeal inflammation was detected in one brain specimen. Similar findings were seen in brains from 43 COVID-19 fatal cases [36]. The commonest finding was reactive microglia (86%), with inflammatory infiltrate by T cytotoxic cells, mainly in the brainstem, cerebellum and leptomeninges. They also detected SARS-CoV-2 in the brain of 53% of the patients, and inflammatory reaction was not associated with SARS-CoV-2 immunohistochemistry positivity (using anti-nucleocapsid protein and anti-spike protein primary antibodies) in the brain. Possible explanations would be that SARS-CoV-2 could infect specific central nervous system compartments; infection may not be associated with local inflammatory reaction, in an immune evasion mechanism, or producing different types of central nervous system involvement [37]. Our series corroborates previous neuropathological studies that disclosed hypoxic injury [35], alterations related to the critical multiorgan failure condition of these patients, such as hemorrhagic foci and splenial lesions, as well as SARS-CoV-2 brain invasion, detected by immunohistochemistry and/or RNA detection.

We can highlight that IHC reactions were positive even in the samplings of brain with normal postmortem MRI.

Pathological–imaging correlation was limited due to the features of postmortem transethmoidal tissue sampling, since it was not possible to conduct a guided sampling of the representative area in a real-time correlation between imaging findings and anatomopathological examination. Thus, some imaging findings may not be represented in pathological samples. However, imaging and pathological findings complemented each other, since MRI included the whole brain (albeit with limited direct viral analysis, spatial resolution, etc.), while pathology was able to examine microscopic details, as well as IH and RT-PCR. In addition, these correlation links in vivo findings (which can be achieved with imaging studies), with pathological analysis (that are only possible with biopsies or postmortem), thus helping establish a better understanding of the in vivo imaging findings.

The sample size of the study was small, so more extensive studies are needed to have a better understanding of the overall neuropathological burden. Future directions include different tissue sampling approaches to try to address all imaging findings in postmortem examination in the autopsy room, additive histological techniques (e.g., electron microscopy and immunofluorescence), as well as extensive brain analysis of patients with different forms and moments of the disease.

## Data Availability

The datasets used and/or analyzed during the current study are available from the corresponding author on reasonable request.

## Abbreviations

ACE2: Angiotensin-converting enzyme 2
ADEM: Acute disseminated encephalomyelitis
COVID-19: Coronavirus disease
ECMO: Extracorporeal membrane oxygenation
IHC: Immunohistochemical
MIA: Minimally invasive autopsy
MIS-C: Multisystem inflammatory syndrome in children
NIHSS: National Institutes of Health Stroke Scale/Score
PRES: Posterior reversible encephalopathy
RNA: Ribonucleic acid
RT-PCR: Reverse transcription polymerase chain reaction
